# The prevalence of anemia and its association with 90-day mortality in hospitalized community-acquired pneumonia

**DOI:** 10.1186/1471-2466-10-15

**Published:** 2010-03-16

**Authors:** Michael C Reade, Lisa Weissfeld, Derek C Angus, John A Kellum, Eric B Milbrandt

**Affiliations:** 1 Department of Intensive Care Medicine, Austin Hospital & University of Melbourne, Melbourne, Victoria, Australia; 2 The CRISMA (Clinical Research, Investigation, and Systems Modeling of Acute Illness) Laboratory, Department of Critical Care Medicine, University of Pittsburgh, Pittsburgh, PA, USA; 3 Department of Biostatistics, University of Pittsburgh Graduate School of Public Health, Pittsburgh, PA, USA

## Abstract

**Background:**

The prevalence of anemia in the intensive care unit is well-described. Less is known, however, of the prevalence of anemia in hospitalized patients with lesser illness severity or without organ dysfunction. Community-acquired pneumonia (CAP) is one of the most frequent reasons for hospitalization in the United States (US), affecting both healthy patients and those with comorbid illness, and is typically not associated with acute blood loss. Our objective was to examine the development and progression of anemia and its association with 90d mortality in 1893 subjects with CAP presenting to the emergency departments of 28 US academic and community hospitals.

**Methods:**

We utilized hemoglobin values obtained for clinical purposes, classifying subjects into categories consisting of no anemia (hemoglobin >13 g/dL), at least borderline (≤ 13 g/dL), at least mild (≤ 12 g/dL), at least moderate (≤ 10 g/dL), and severe (≤ 8 g/dL) anemia. We stratified our results by gender, comorbidity, ICU admission, and development of severe sepsis. We used multivariable logistic regression to determine factors independently associated with the development of moderate to severe anemia and to examine the relationship between anemia and 90d mortality.

**Results:**

A total of 8240 daily hemoglobin values were measured in 1893 subjects. Mean (SD) number of hemoglobin values per patient was 4.4 (4.0). One in three subjects (33.9%) had at least mild anemia at presentation, 3 in 5 (62.1%) were anemic at some point during their hospital stay, and 1 in 2 (54.5%) survivors were discharged from the hospital anemic. Anemia increased with illness severity and was more common in those with comorbid illnesses, female gender, and poor outcomes. Yet, even among men and in those with no comorbidity or only mild illness, anemia during hospitalization was common (~55% of subjects). When anemia was moderate to severe (≤ 10 g/dL), its development was independently associated with increased 90d mortality, even among hospital survivors.

**Conclusions:**

Anemia was common in hospitalized CAP and independently associated with 90d mortality when hemoglobin values were 10 g/dL or less. Whether prevention or treatment of CAP-associated anemia would improve clinical outcomes remains to be seen.

## Background

The prevalence of anemia in the intensive care unit (ICU) is well-described [1-4]. In this setting, anemia is common, increasing during the hospital stay, and associated with poor outcomes. Less is known, however, of the prevalence of anemia in hospitalized patients with lesser illness severity or without organ dysfunction [5,6]. There is increasing interest in manipulating hemoglobin values in hospitalized patients with interventions such as red blood cell transfusions [7], recombinant human erythropoetin [8], and blood substitutes [9]. If these or other as yet undiscovered therapies were proven to improve patient outcomes, their application to clinical practice would require a much better understanding of anemia in both ICU and non-ICU patients.

Community-acquired pneumonia (CAP) is one of the most frequent causes of hospitalization in the United States (US), affecting both otherwise healthy patients and those with comorbid illness. In patients hospitalized with CAP, studies show that anemia is common and associated with increased length of stay and mortality [10-18]. Yet, most studies were small, retrospective, or limited to a single center. Existing large studies primarily examined hemoglobin values at presentation [10,13,14] and therefore miss the evolution of changes in hemoglobin values over the course of hospitalization. Furthermore, no study has focused exclusively on anemia in CAP, its course over time, and its prevalence across important subgroups to understand the independent contribution of illness severity, comorbidty, and gender on the development of anemia.

We therefore examined the development and progression of anemia in a large, prospective, multicenter inception cohort study of subjects presenting to the emergency department with CAP, a primarily non-critically ill cohort with less than 1 in 6 subjects admitted to an ICU. Our primary goals were to describe the prevalence of anemia at presentation and its development over time, not only in those previously at risk of anemia, but also in those without common anemia risk factors. Furthermore, we sought to determine whether anemia is independently associated with increased 90d mortality. We hypothesized that anemia would increase with illness severity and be greater in those with comorbid illnesses, female gender, and poor outcomes.

## Methods

### Sites and subjects

The Genetic and Inflammatory Markers of Sepsis (GenIMS) study enrolled subjects at 28 academic and community hospitals in southwestern Pennsylvania, Connecticut, southern Michigan, and western Tennessee from December 2001 and November 2003. GenIMS included patients ≥ 18 years old with a clinical and radiologic diagnosis of pneumonia, as per the criteria of Fine et al [13]. We excluded: transfer from another hospital; discharge from a hospital within the prior 10 days; an episode of pneumonia within the prior 30 days; chronic mechanical ventilation, cystic fibrosis, or active pulmonary tuberculosis; admission for palliative care; previous enrollment in the study; incarceration; and pregnancy. For the purposes of this GenIMS substudy, we excluded subjects who had no hemoglobin measurement during their hospital stay. Participants or their proxies provided written consent. We obtained approval from the Institutional Review Boards of the University of Pittsburgh and all participating sites. Other results of this study, not inclusive of these anemia data, have been published elsewhere [19,20].

### Clinical definitions and outcome variables

We prospectively collected detailed baseline and sequential clinical and laboratory information using structured subject or proxy interviews, bedside assessments, and medical record abstraction. We obtained all hemoglobin values performed for clinical purposes from the medical record. The World Health Organization defines anemia as hemoglobin levels <13 g/dL in men or <12 g/dL in women [21]. Because we wished to explore the prevalence and significance of anemia of varying degrees of severity, we classified anemia based on hemoglobin values into categories consisting of no anemia (>13 g/dL), at least borderline (≤ 13 g/dL), at least mild (≤ 12 g/dL), at least moderate (≤ 10 g/dL), and severe (≤ 8 g/dL) anemia. The hemoglobin value obtained on day 1 was defined as the baseline "hemoglobin on presentation" value. The final hemoglobin measured in the hospital was the discharge value.

We ascertained comorbid conditions using the Charlson comorbidity index [22] and severity of illness using APACHE III [23] and the Pneumonia Severity Index (PSI) [13]. We defined severe sepsis as pneumonia plus acute organ dysfunction following the 2001 International Consensus Criteria [24]. We defined acute organ dysfunction as a new Sequential Organ Failure Assessment (SOFA) [25] score of ≥ 3 in any of six organ systems, based on the international Sepsis Occurrence in the Acutely ill Patient study [26]. We determined survival post-discharge by telephone and National Death Index search. We used 90-day mortality as our primary measure of survival, based on endpoint recommendations for sepsis trials from two recent international expert panels [27,28].

### Statistical analysis

Statistical analyses were performed using SAS software, version 9.1 (SAS Institute, Cary, NC), with statistical significance set at p < 0.05. We compared differences for single points in time using chi-square test or Fisher's exact test for dichotomous data and Student's t-test or Mann-Whitney U for continuous data. For analyses in which a baseline value was necessary (determining the prevalence of anemia on presentation and stratifying the subsequent course of hemoglobin values by presenting hemoglobin category), we limited our analysis to those subjects with day 1 hemoglobin values obtained (n = 1838). For analyses not requiring a baseline value (determining the prevalence of anemia during hospitalization, risk factors for its development, and the association with 90d mortality), we included all subjects who had hemoglobin measured at least once during their hospital stay (the "inpatient CAP analysis cohort", n = 1893).

To determine variables independently associated with the development of moderate to severe anemia in subjects hospitalized with CAP, we conducted multivariable logistic regression using backward stepwise variable selection with p < 0.05 for model retention. Variables eligible for inclusion in the model were baseline characteristics (age, gender, race, Charlson comorbidity, chronic cardiac or respiratory disease, cirrhosis, and chronic hemodialysis), day 1 severity of illness (APACHE III, PSI, SOFA), and clinical course (development of severe sepsis, ICU admission, and use of mechanical ventilation). The same variables were used for modeling 90d mortality, but also included the development of moderate to severe anemia and whether a subject received a blood transfusion during their hospital stay. For both models, we conducted our primary analyses in the entire inpatient CAP analysis cohort, with secondary analysis of the 90d mortality model limited to subjects who survived to hospital discharge.

## Results

### Study population and outcomes

**Figure 1:**
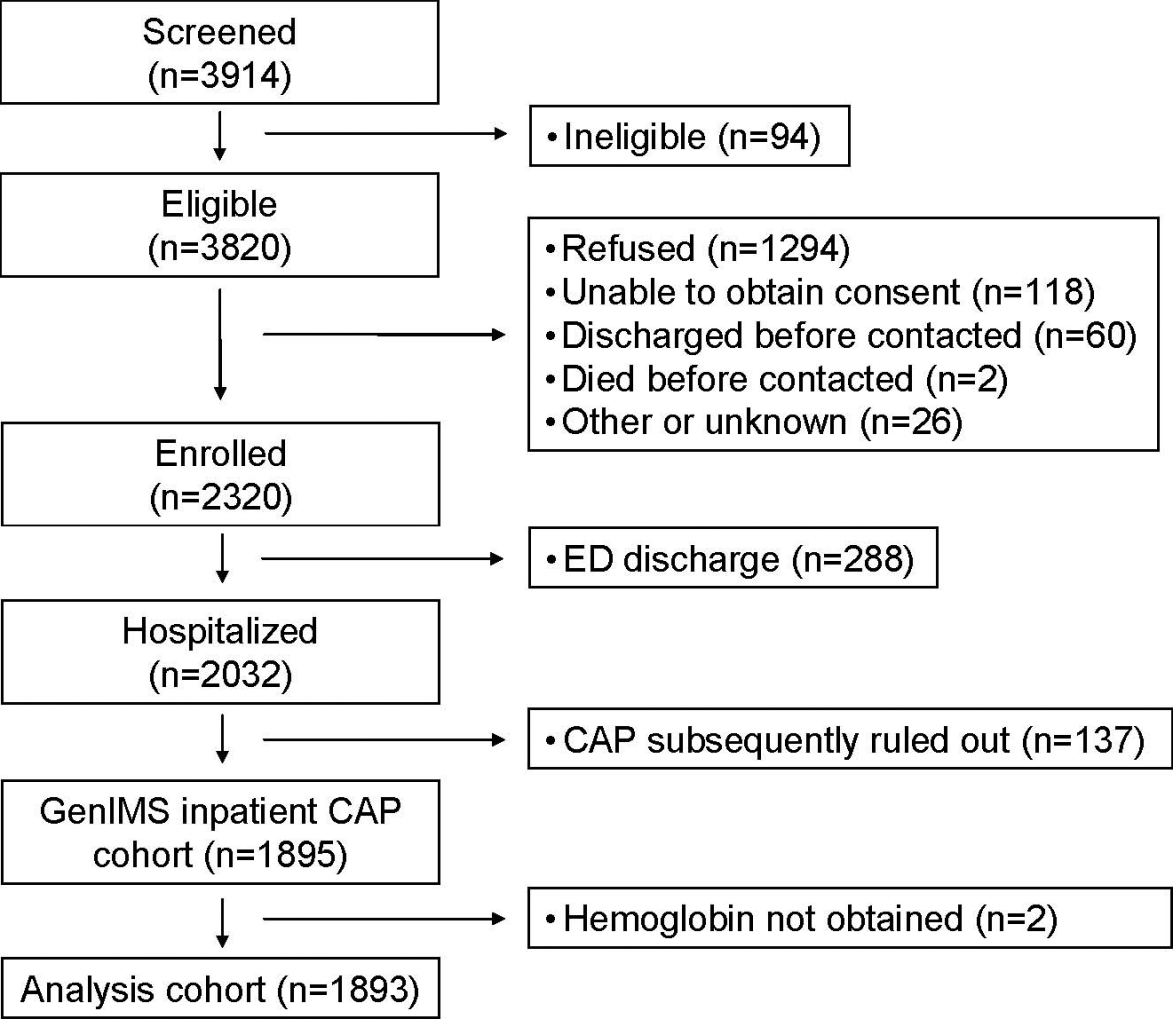
**Flow diagram for the entire GenIMS cohort**.

Of 2320 enrolled subjects, we excluded 288 (12%) who were discharged from the emergency department and 137 (6%) because their treating physicians subsequently ruled out pneumonia as the cause of their illness. The remaining 1895 subjects comprised the inpatient CAP cohort (figure 1 and table 1). In this group, 1838 (97.0%) had hemoglobin values obtained on day 1 and recorded in the medical record, while all but two had hemoglobin values obtained at least once during their stay, leaving 1893 (99.7%) in the analysis cohort. A total of 8240 daily hemoglobin values were measured. Mean (SD) number of hemoglobin values per patient was 4.4 (4.0), with median (interquartile range) 3 (2 to 5). Mean (SD) hospital length of stay was 7.3 (5.0) days. After day 1, hemoglobin values were available for a mean (SD) of 55.3% (7.3%) subjects who remained hospitalized each day. Of the 1893 in the analysis cohort, 114 (6.0%) had positive sputum cultures, 139 (7.3) were bacteremic, 582 subjects (30.7%) developed severe sepsis, 124 (6.6%) died within 30d of enrollment, and 215 (11.4%) died within 90d of enrollment.

**Table 1:** Clinical characteristics at baseline and during the study

			ICU Admission		Severe Sepsis		Comorbidity	
		All	Yes	No	Yes	No	Yes	No
N		1893	303 (16.0)	1590 (84.0)	582 (30.7)	1311 (69.3)	1373 (72.5)	520 (27.5)
Age, years, mean (SD)		67.8 (16.8)	68.1 (15.7)	67.7 (17.0)	71.5 (15.7)	66.1 (17.0)	69.9 (15.3)	62.2 (19.1)
Male gender, n (%)		983 (51.9)	168 (55.4)	815 (51.3)	329 (56.5)	654 (49.9)	738 (53.8)	245 (47.1)
Race, n (%)								
White		1527 (80.7)	245 (80.9)	1282 (80.6)	494 (84.9)	1033 (78.8)	1121 (81.6)	406 (78.1)
Black		299 (15.8)	51 (16.8)	248 (15.6)	73 (12.5)	226 (17.2)	206 (15.0)	93 (17.9)
Other		67 (3.5)	7 (2.3)	60 (3.8)	15 (2.6)	52 (4.0)	46 (3.4)	21 (4.0)
Underlying disease^a^								
Charlson comorbidity >0, n (%)		1373 (72.5)	225 (74.3)	1148 (72.2)	446 (76.6)	927 (70.7)	1373 (100.0)	n/a
Charlson comorbidity, mean (SD)		1.9 (2.2)	1.9 (2.2)	1.9 (2.2)	2.2 (2.3)	1.8 (2.2)	2.7 (2.2)	n/a
Cardiovascular disease, n (%)		487 (25.7)	79 (26.1)	408 (25.7)	163 (28.0)	324 (24.7)	487 (35.5)	n/a
Respiratory disease, n (%)		717 (37.9)	118 (38.9)	599 (37.7)	207 (35.6)	510 (38.9)	717 (52.2)	n/a
Chronic hemodialysis, n (%)		48 (2.5)	11 (3.6)	37 (2.3)	9 (1.5)	39 (3.0)	48 (3.5)	n/a
Cirrhosis, n (%)		5 (0.3)	1 (0.3)	4 (0.3)	1 (0.2)	4 (0.3)	5 (0.4)	n/a
PSI, mean (SD)		99.8 (38.1)	124.2 (40.7)	95.1 (35.8)	121.2 (40.4)	90.3 (32.9)	106.8 (37.8)	81.4 (32.3)
PSI class, n (%)								
I and II		428 (22.6)	26 (8.6)	402 (25.3)	55 (9.5)	373 (28.5)	216 (15.7)	212 (40.8)
III		392 (20.7)	35 (11.6)	357 (22.5)	76 (13.1)	316 (24.1)	265 (19.3)	127 (24.4)
IV		708 (37.4)	116 (38.3)	592 (37.2)	230 (39.5)	478 (36.5)	571 (41.6)	137 (26.3)
V		365 (19.3)	126 (41.6)	239 (15.0)	221 (38.0)	144 (11.0)	321 (23.4)	44 (8.5)
APACHE III, mean (SD)		56.1 (17.9)	67.7 (20.5)	53.9 (16.4)	65.8 (19.8)	51.8 (15.1)	58.6 (17.6)	49.4 (16.8)
SOFA, mean (SD)		2.4 (1.9)	3.7 (2.7)	2.2 (1.7)	3.7 (2.4)	1.8 (1.3)	2.5 (2.1)	2.1 (1.6)
Duration of symptoms prior to ED presentation, days^b^, mean (SD)		4.9 (7.1)	4.7 (6.8)	5.0 (7.2)	4.9 (7.6)	5.0 (6.9)	4.8 (7.2)	5.3 (7.0)
Antibiotics before presentation, n (%)		333 (17.6)	57 (18.8)	276 (17.4)	93 (16.0)	240 (18.3)	233 (17.0)	100 (19.2)
Transfusion in hospital, n (%)		176 (9.3)	97 (32.0)	79 (5.0)	125 (21.5)	51 (3.9)	138 (10.1)	38 (7.3)
ICU admission, n (%)		303 (16.0)	303 (100.0)	n/a	225 (38.7)	78 (5.9)	225 (16.4)	78 (15.0)
Mechanical ventilation, n (%)		132 (7.0)	123 (40.6)	9 (0.6)	132 (22.7)	0 (0.0)	89 (6.5)	43 (8.3)
Hospital length of stay, days, mean (SD)		7.3 (5.0)	12.7 (7.2)	6.3 (3.6)	10.3 (6.7)	6.0 (3.3)	7.5 (4.9)	7.0 (5.3)
Hospital mortality, n (%)		87 (4.6)	53 (17.5)	34 (2.1)	83 (14.3)	4 (0.3)	71 (5.2)	16 (3.1)
90-day mortality, n (%)		215 (11.4)	80 (26.4)	135 (8.5)	150 (25.8)	65 (5.0)	184 (13.4)	31 (6.0)

PSI, Pneumonia Severity Index; SOFA, Sequential Organ Failure Assessment; APACHE III, Acute Physiology and Chronic Health Evaluation III score; ED, Emergency Department; ICU, intensive care unit.

^a^According to the method of Charlson et al [22]

^b ^Missing for 19 subjects

### Anemia at presentation

Among the 1838 subjects with day 1 measurements, mean (SD) hemoglobin was 12.8 (1.9) g/dL. One in three (33.9%) subjects had at least mild anemia (hemoglobin ≤ 12 g/dL) at presentation (table 2). This was true even in men, in those with no prior history of chronic disease, and in those without acute organ dysfunction or requiring ICU care. More severe forms of anemia were less common, with 8.1% and 1.1% having at least moderate (≤ 10 g/dL) or severe (≤ 8 g/dL) anemia at presentation. In general, each level of anemia at presentation was significantly more common in women, those with comorbidities, and the more severely ill.

**Table 2:** Hemoglobin on day 1, during hospitalization, and at discharge in the GenIMS cohort.

			ICU Admission		Severe Sepsis		Comorbidity		Gender	
		All	Yes	No	Yes	No	Yes	No	Female	Male
Hemoglobin on day 1^a^, n (%)										
No anemia (hgb >13 g/dL)		857 (46.6)	132 (44.4)	725 (47.0)	234 (41.5)	623 (48.9)*	593 (44.5)	264 (52.2)*	331 (37.5)	526 (55.0)*
At least borderline (hgb ≤ 13 g/dL)		981 (53.4)	165 (55.6)	816 (53.0)	330 (58.5)	651 (51.1)*	739 (55.5)	242 (47.8)*	551 (62.5)	430 (45.0)*
At least mild (hgb ≤ 12 g/dL)		624 (33.9)	115 (38.7)	509 (33.0)	232 (41.1)	392 (30.8)*	481 (36.1)	143 (28.3)*	353 (39.9)	272 (28.5)*
At least moderate (hgb ≤ 10 g/dL)		149 (8.1)	43 (14.5)	106 (6.9)*	74 (13.1)	75 (5.9)*	126 (9.5)	23 (4.5)*	82 (9.3)	67 (7.0)
Severe (hgb ≤ 8 g/dL)		20 (1.1)	9 (3.0)	11 (0.7)*	10 (1.8)	10 (0.8)	17 (1.3)	3 (0.6)	17 (1.9)	3 (0.3)*
Hemoglobin during hospital stay^b^, n (%)										
No anemia (hgb >13 g/dL)		407 (21.5)	36 (11.9)	371 (23.3)*	89 (15.3)	318 (24.3)*	280 (20.4)	127 (24.4)	133 (14.6)	274 (27.9)*
At least borderline (hgb ≤ 13) g/dL		1486 (78.5)	267 (88.1)	1219 (76.7)*	493 (84.7)	993 (75.7)*	1093 (79.6)	393 (75.6)	777 (85.4)	709 (72.1)*
At least mild (hgb ≤ 12 g/dL)		1175 (62.1)	244 (80.5)	931 (58.6)*	430 (73.9)	745 (56.8)*	879 (64.0)	296 (56.9)*	639 (70.2)	536 (54.5)*
At least moderate (hgb ≤ 10 g/dL)		511 (27.0)	170 (56.1)	341 (21.4)*	259 (44.5)	252 (19.2)*	388 (28.3)	123 (23.7)*	275 (30.2)	236 (24.0)*
Severe (hgb ≤ 8 g/dL)		93 (4.9)	50 (16.5)	43 (2.7)*	59 (10.1)	34 (2.6)*	67 (4.9)	26 (5.0)	53 (5.8)	40 (4.1)
Hemoglobin at discharge in survivors^c^, n (%)										
No anemia (hgb >13 g/dL)		473 (26.2)	44 (17.6)	429 (27.6)*	100 (20.0)	373 (28.5)*	324 (24.9)	149 (29.6)*	161 (18.4)	312 (33.4)*
At least borderline (hgb ≤ 13) g/dL		1333 (73.8)	206 (82.4)	1127 (72.4)*	399 (80.0)	934 (71.5)*	978 (75.1)	355 (70.4)*	712 (81.6)	621 (66.6)*
At least mild (hgb ≤ 12 g/dL)		982 (54.4)	171 (68.4)	811 (52.1)*	322 (64.5)	660 (50.5)*	729 (56.0)	253 (50.2)*	552 (63.2)	430 (46.1)*
At least moderate (hgb ≤ 10 g/dL)		286 (15.8)	56 (22.4)	230 (14.8)*	113 (22.6)	173 (13.2)*	213 (16.4)	73 (14.5)	164 (18.8)	122 (13.1)*
Severe (hgb ≤ 8 g/dL)		16 (0.9)	3 (1.2)	13 (0.8)	5 (1.0)	11 (0.8)	10 (0.8)	6 (1.2)	12 (1.4)	4 (0.4)*

Hgb, hemoglobin; ICU, intensive care unit

* p < 0.05 for within group comparison

^a ^N = 1838 had day 1 hemoglobin measured

^b ^N = 1893 had hemoglobin values obtained at least once during their stay

^c ^N = 1806 hospital survivors

### Anemia over time

**Figure 2:**
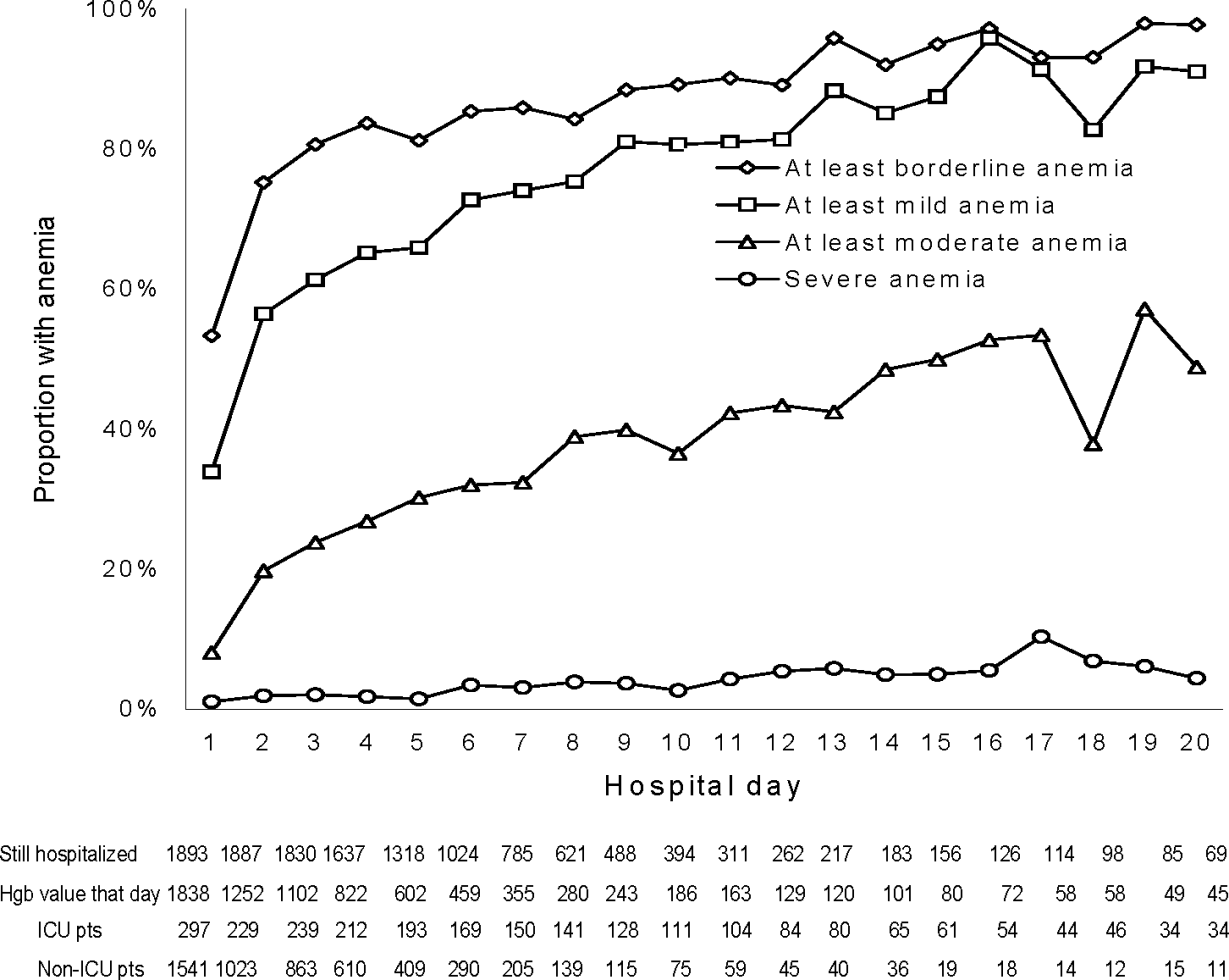
**Prevalence of anemia by day for patients remaining in hospital on each day and having a hemoglobin obtained that day**. ICU patients refers to those who spent at least one day in the ICU. Non-ICU patients were never admitted to the ICU. During the first week, the majority of hemoglobin values were obtained from non-ICU patients. After day 7, those still remaining in the hospital and providing daily hemoglobin values were more likely to have spent at least one day of their hospital stay in the ICU. Despite this, 2/3 (341 of 511) of those with moderate to severe anemia were non-ICU patients.

**Figure 3:**
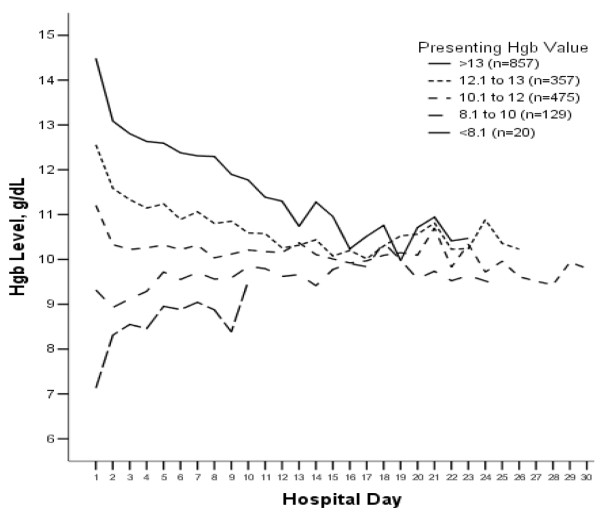
**Course of hemoglobin by presenting hemoglobin category for patients remaining in hospital and having a hemoglobin obtained that day**. Regardless of presenting hemoglobin category, mean hemoglobin concentrations regressed to a common value of around 10 g/dl. Censored for <5 observations/category/day to reduce effect of outliers. N = 55 subjects with no day 1 hemoglobin measurement excluded.

Three in five (62.1%) subjects developed at least mild anemia at some point during their hospital stay (table 2), most of which occurred by the second hospital day. Anemia was more common in women, those with comorbidities, and the more severely ill, yet was often present in those without these risk factors. For those remaining in the hospital, anemia became progressively more common with each passing day (figure 2). Over time, hemoglobin values converged to around 10 g/dL, regardless of the presenting hemoglobin value (figure 3). Of all subjects, 176 (9.3%) received at least one blood transfusion. Of those who were transfused, mean (SD) pre-transfusion hemoglobin was 8.5 (1.2) g/dL.

### Anemia at hospital discharge

**Figure 4:**
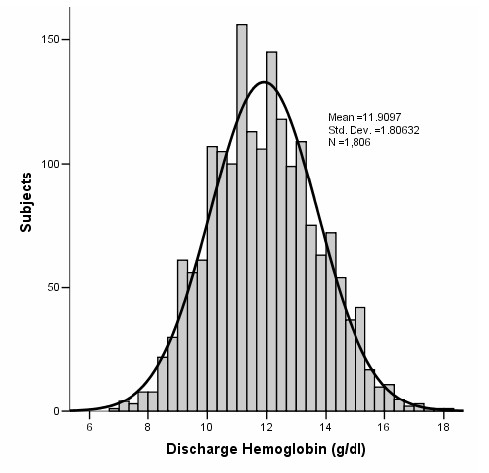
**Discharge hemoglobin values among hospital survivors**. For this graph, we analyzed the last hemoglobin measurement in subjects discharged alive from the hospital (n = 1806). Hemoglobin was measured on the day of discharge in 512 (28.3%) subjects and within 48 hours before discharge in an additional 795 (44%) subjects. For a minority of subjects (n = 164, 9%), the last available hemoglobin concentration measurement was more than 96 hours before discharge. Mean (SD) discharge hemoglobin was 11.9 (1.8) g/dL, indicating that the average hospital survivor had mild anemia at discharge.

Of the 1893 subjects in the analysis cohort, 1806 (95.4%) survived to hospital discharge. Hemoglobin was measured on the day of discharge in 512 (28.3%) subjects and within 48 hours before discharge in an additional 795 (44%) subjects. For a minority of subjects (n = 164, 9%), the last available hemoglobin concentration measurement was more than 96 hours before discharge. Mean (SD) discharge hemoglobin was 11.9 (1.8) g/dL (figure 4), indicating that the average hospital survivor had mild anemia at discharge. More than half (54.4%) of survivors were sent home with at least mild anemia (table 2), including 63% of women and 46% of men. Of hospital survivors with at least two measurements during their stay, hemoglobin dropped a mean (SD) of 1.1 (1.3) g/dL.

### Variables independently associated with development of moderate to severe anemia

In multivariable modeling, we explored risk factors for the development of moderate to severe anemia (hemoglobin ≤ 10 g/dL) (table 3). Female gender, comorbidity, initial illness severity, and measures of clinical course were independently associated with moderate to severe anemia. White race and the presence of chronic respiratory disease were inversely associated with moderate to severe anemia. These latter two observations did not appear to be due differences in the incidence or number of units of blood transfused, neither of which were greater in these groups (data not shown).

**Table 3:** Variables independently associated with development of moderate to severe anemia.

Variable	Adj OR	95% CI		P-value
Age	0.99	0.979	0.998	0.022
Male	0.46	0.36	0.59	<0.001
Race				0.001
White	ref			
Black	1.86	1.33	2.60	
Other	1.43	0.76	2.69	
Charlson comorbidity	1.10	1.04	1.16	0.001
Chronic respiratory disease	0.46	0.35	0.59	<0.001
APACHE III	1.02	1.01	1.03	<0.001
PSI	1.01	1.01	1.02	<0.001
Severe sepsis	1.40	1.06	1.85	0.017
ICU admission	1.79	1.25	2.55	0.001
Mechanical ventilation	3.57	2.07	6.15	<0.001
Constant	0.14			<0.001

ICU, intensive care unit; Adj OR, adjusted odds ratio; CI, confidence interval; APACHE III, Acute Physiology and Chronic Health Evaluation III score; PSI, Pneumonia Severity Index.

### Anemia and its association with 90d mortality

After adjusting for baseline characteristics, initial illness severity, and measures of clinical course, the development of moderate to severe anemia (hemoglobin ≤ 10 g/dL) at any time during the hospital stay was independently associated with increased 90d mortality (adjusted odds ratio [95% confidence interval]: 1.59 [1.12 to 2.25], p = 0.01) (table 4). This association persisted when limiting our analysis to subjects who survived to hospital discharge (1.64 [1.09 to 2.47], p = 0.02) (table 5). In neither model was occurrence of no more than mild anemia or the receipt of a blood transfusion independently associated with mortality. The latter was true even if moderate to severe anemia was excluded from the model (data not shown).

**Table 4:** Multivariable logistic regression model for 90d mortality.

Variable	Adj OR	95% CI		P-value
Age	1.04	1.02	1.05	<0.001
Charlson	1.12	1.04	1.20	0.002
Cardiac disease	0.64	0.44	0.93	0.020
Cirrhosis	16.58	1.98	139.09	0.010
PSI	1.02	1.01	1.02	<0.001
Severe sepsis	3.00	2.07	4.35	<0.001
Mechanical ventilation	2.69	1.61	4.48	<0.001
Ever moderate to severe anemia	1.59	1.12	2.25	0.010
Constant	0.001			<0.001

CI, confidence interval; PSI, Pneumonia Severity Index.

**Table 5:** Multivariable logistic regression model for 90d mortality for hospital survivors.

Variable	Adj OR	95% CI		P-value
Age	1.04	1.02	1.05	<0.001
Charlson	1.13	1.04	1.22	0.003
Cirrhosis	17.27	2.05	145.64	0.009
PSI	1.01	1.005	1.02	0.001
Severe sepsis	1.97	1.31	2.97	0.001
Ever moderate to severe anemia	1.64	1.09	2.47	0.018
Constant	0.001			<0.001

CI, confidence interval; PSI, Pneumonia Severity Index.

## Discussion

Anemia was surprisingly common in hospitalized CAP, increasing with illness severity and greater in those with comorbid illnesses, female gender, and poor outcomes. Yet, even among men and in those with no comorbidity or only mild illness, anemia was extremely common. One in three subjects had at least mild anemia at presentation, 3 in 5 were anemic at some point during their hospital stay, and 1 in 2 were sent home anemic. When anemia was moderate to severe (hemoglobin ≤ 10 g/dL), its development was independently associated with increased 90d mortality, even among hospital survivors and after accounting for illness severity.

The prevalence of anemia in hospitalized CAP depends upon the definition used. The World Health Organization defines anemia as hemoglobin levels <13 g/dL in men or <12 g/dL in women [21]. According to these criteria, anemia is prevalent in 2.9% of men and 7.5% of women in the general US population [29], rising to around 7-9% in the elderly [29,30]. Using a conservative cutoff of 12 g/dL for both genders, the prevalence of anemia seen in our study was considerably higher than that of the general population. Given the known associations of WHO-defined anemia with increased mortality [31], as well as poor physical and cognitive function [32], our findings offer cause for concern.

Previous large inpatient CAP cohort studies defined anemia as a hemoglobin of <10 g/dL or hematocrit <30%, yielding a prevalence of anemia on presentation of 7-12% [10,13,14], proportions which are consistent with our own. Our study is unique, in that we were able to describe not only the prevalence of anemia on presentation, but its development over time and its persistence at the time of hospital discharge. Furthermore, rather than focusing on arbitrary cutoffs, we explored the full spectrum of anemia severity across important subgroups, and in doing so, can better understand the prevalence and significance of anemia in hospitalized CAP and the independent contribution of patient and illness characteristics to its development.

Why was anemia so common in hospitalized CAP, even among those without obvious risk factors? The day 1 prevalence speaks to the significant systemic derangement that likely has already occurred in CAP patients at the time of presentation, long before repeated blood draws or the dilutional effects of intravenous fluids could explain low hemoglobin levels. The precipitous decline in hemoglobin values that occurred over the first few days of hospitalization is consistent with that seen in the ICU, where hemoglobin values may decline by >0.5 g/dL/day in non-bleeding patients [33,34]. These changes are believed to be due not only to dilutional effects of fluids and frequent blood draws [6], but also to other sources of blood loss (gastric stress bleeding, surgical procedures), effects of inflammatory cytokines, inadequate red cell production, and excessive red cell destruction [35]. Unfortunately, the nature of the data collected in our study precluded us from identifying which of these factors were at work in any given patient. Certain infections, such as *Mycoplasma pneumonia*, are associated with anemia. Very few subjects in our study had positive blood or sputum cultures and cultures were not universally drawn, which is typical for observational studies of CAP [14,36]. Consequently, we could not reliably determine whether the prevalence or severity of anemia varied by presence of bacteremia or by type of infecting organism.

In our study, the presence of moderate to severe anemia was independently associated with increased 90d mortality after accounting for factors such as comorbidity, initial illness severity, the development of severe sepsis, and use of mechanical ventilation. This association persisted when limited to hospital survivors. Other studies of CAP patients have identified anemia as a risk factor for mortality [11,15,17,18] and an initial hematocrit level of <30% is a component of the Pneumonia Severity Index [13], which classifies 30d mortality risk. Yet, does the anemia actually cause increased mortality or is it merely an additional marker of illness severity, and therefore, mortality?

In chronic disease states, inflammatory cytokines are thought to contribute to the development of anemia by shortening red blood cell survival and impairing ability of red blood cell progenitors to respond to erythropoietin [37]. Despite clinical recovery, many patients with CAP leave the hospital with ongoing subclinical inflammation, which is associated with an increased risk of death due to cardiovascular disease, cancer, infections, and renal failure [38]. If a proinflammatory state is associated with both the development of anemia and mortality, might anemia be in the causal pathway between inflammation and mortality? Observational studies like our own cannot answer this question, yet interventional studies suggest that while anemia is bad, correcting it not necessarily helpful and perhaps, deleterious [3,8].

There are important caveats and limitations to our work that deserve consideration. We did not have access to pre-CAP hemoglobin levels, and were therefore unable to determine if anemia preceded the development of pneumonia. Yet anemia was common even among those without well-known risk factors for chronic anemia. Hemoglobin values were only available if drawn for clinical purposes, which occurred in 97% of subjects on day 1, but in slightly more than half of those remaining in the hospital on each subsequent day. Consequently, though our anemia prevalence rates for day 1 and the entire hospital stay are accurate; our day-specific rates (figure 2) may be either over or underestimated depending on hemoglobin values for those who were not sampled that day. The prevalence of anemia among inpatients increased over the hospital stay in part due to discharge of less severely ill patients, with the remaining patients subject to the hemoglobin lowering effects of additional blood draws. Even so, the prevalence of anemia in those who were discharged was quite high. We are unable to explain why chronic respiratory disease appeared protective for the development of anemia in our models when it is known to be a risk factor for anemia in the outpatient setting [37]. Importantly, this observation did not seem to be due differential transfusion rates in our cohort. It was impractical to draw blood samples after hospital discharge; therefore our results only describe hemoglobin changes during the hospital stay. Whether anemia persists after hospital discharge remains to be seen. If so, this might present a unique time to intervene if persistent abnormalities were also associated with adverse outcomes [39].

## Conclusions

Anemia was common in hospitalized CAP, not only in those with severe illness or anemia risk factors, but also in those with mild illness and no risk factors. When moderate to severe (hemoglobin ≤ 10 g/dL), the development of anemia was independently associated with increased 90d mortality. Whether prevention or treatment of CAP-associated anemia would improve clinical outcomes remains to be seen.

## Competing interests

DCA received consulting fees previously from OrthoBiotech and Amgen, but has not received any fees in the last five years and has no ongoing financial relationships related to this work. The remaining authors declare that they have no commercial association or financial involvement that might pose a conflict of interest in connection with this article.

## Authors' contributions

MCR, DCA, JAK, and EBM contributed to the conception and design, acquisition of data, analysis and interpretation of data, drafting and revising the manuscript. LW contributed to the conception and design, acquisition of data, and analysis and interpretation of data. All authors provided final approval of the version to be published.

## Pre-publication history

The pre-publication history for this paper can be accessed here:

http://www.biomedcentral.com/1471-2466/10/15/prepub
